# Lestaurtinib Has the Potential to Inhibit the Proliferation of Hepatocellular Carcinoma Uncovered by Bioinformatics Analysis and Pharmacological Experiments

**DOI:** 10.3389/fcell.2022.837428

**Published:** 2022-05-13

**Authors:** Shuang Wu, Shihai Liu, Yan Li, Changchang Liu, Huazheng Pan

**Affiliations:** ^1^ Department of Clinical Laboratory, The Affiliated Hospital of Qingdao University, Qingdao, China; ^2^ Department of Medicine, Qingdao University, Qingdao, China; ^3^ Medical Animal Laboratory, The Affiliated Hospital of Qingdao University, Qingdao, China; ^4^ Department of Operation Room, The Affiliated Hospital of Qingdao University, Qingdao, China

**Keywords:** hepatocellular carcinoma, lestaurtinib, bioinformatics analysis, STAT family, pharmacological experiments

## Abstract

Patients diagnosed with hepatocellular carcinoma (HCC) seek a satisfactory prognosis. However, most HCC patients present a risk of recurrence, thus highlighting the lack of effectiveness of current treatments and the urgent need for improved treatment options. The purpose of this study was to identify new candidate factors in the STAT family, which is involved in hepatocellular carcinogenesis, and new targets for the treatment of HCC. Bioinformatics web resources, including Oncomine, Gene Expression Profiling Interactive Analysis (GEPIA), The Human Protein Atlas (HPA), Tumor Immune Estimation Resource (TIMER), and GSCALite, were used to identify candidate genes among the STAT family in HCC. STAT1 was significantly overexpressed in hepatocellular carcinoma. More meaningfully, the high STAT1 expression was significantly associated with poor prognosis. Therefore, STAT1 is expected to be a therapeutic target. The JAK2 inhibitor lestaurtinib was screened by the Genomics of Cancer Drug Sensitivity Project (GDSC) analysis. Pharmacological experiments showed that lestaurtinib has the ability to prevent cell migration and colony formation from single cells. We also found that STAT1 is involved in inflammatory responses and immune cell infiltration. Immune infiltration analysis revealed a strong association between STAT1 levels and immune cell abundance, immune biomarker levels, and immune checkpoints. This study suggests that STAT1 may be a key oncogene in hepatocellular carcinoma and provides evidence that the JAK2 inhibitor lestaurtinib is a potent antiproliferative agent that warrants further investigation as a targeted therapy for HCC.

## Introduction

Hepatocellular carcinoma (HCC) is the sixth-most commonly diagnosed cancer and the third leading cause of cancer death worldwide (Sung et al., 2021). The global incidence of HCC has been increasing, with an estimated range of 600,000–800,000 new cases occurring annually (Sia et al., 2017). Generally, the dominant risk factors for HCC in high–incidence rate countries such as those in Asia and Africa are hepatitis B virus (HBV) infections and aflatoxin B exposure, whereas hepatitis C virus (HCV) infections, alcohol consumption, and metabolic syndromes are more important risk factors in low-incidence regions, which include countries in Europe, North and South America, and the Middle East (Mak et al., 2018; Yang J. D. et al., 2019). HCC has high proliferation, invasion and metastasis potential, low survival rates, and high mortality (Llovet et al., 2021). A large number of studies have discussed the mechanisms underlying the occurrence, recurrence and metastasis, tumorigenesis, and treatment of HCC. However, many questions remain to be clarified (Zhu and Sun, 2019). Patients who lack typical clinical manifestations and typical genotyping cannot be accurately selected for treatment schemes (Kim et al., 2019). Finding the initial driving factor, identifying promising prognostic biomarkers for clinical diagnosis, and revealing targets for drug design are urgent problems that need to be solved.

Increasing evidence has been obtained for the regulation of Janus kinase/transducer and activator of transcription JAK/STAT signaling cytokines and the action of interferons (IFNs), which affect gene expression. In the development of HCC, JAK/STAT signaling is abnormally activated, resulting in the imbalance of these downstream target genes controlling survival, angiogenesis, stem cells, immune monitoring, invasion, and metastasis (Hin Tang et al., 2020). The activating mutations of JAK/STAT signaling or members of cellular biological process, inflammation, and immunity of cancer cells indicate that they are promising biomarkers for drug exploitation and malignancy treatment (Banerjee et al., 2017; Owen et al., 2019; Hin Tang et al., 2020).

A total of seven members of the STAT family have been identified in mammals, namely, STAT1/2/3/4/5A/5B/6. According to the reports in the literature, mutations in the JAK-STAT pathway lead to dysfunction and tumorigenesis. Interestingly, STAT1 and STAT3 were the most common mutations, and they also caused changes in immune function, including aberrant cytokine signaling and responsiveness, poor Th1 differentiation, IFN imbalance, NK cytotoxicity, and loss of autoimmunity (Owen et al., 2019). The STAT family was proposed as a biomarker or immune checkpoint inhibitor for prognosis prediction or therapy in various types of solid tumors, including STAT3 and STAT5A in breast cancer (Wu et al., 2017); STAT3, STAT5A, and STAT6 in lung cancer (Pastuszak-Lewandoska et al., 2017); and STAT3 and STAT5A in prostate cancer (Mohanty et al., 2017). However, the specific functions of the STAT family in liver hepatocellular carcinoma (LIHC) have not yet been systematically described.

In our study, we performed a comprehensive analysis of the expression and genetic alterations of members of the STAT family in HCC and constructed a nomogram that integrates the clinical characteristics of the STAT family. Finally, the effect of the JAK2 inhibitor lestaurtinib on the viability of liver cancer cells was evaluated by pharmacological experiments. Moreover, the association between STAT1 and immune cells, biomarkers, and immune checkpoints of STAT1 in LIHC was also explored. STAT1 is an important factor in the occurrence and development of hepatocellular carcinoma. Based on this, our study found that lestaurtinib has the potential to inhibit the proliferation of hepatocellular carcinoma and is expected to become a new therapeutic drug for HCC patients.

## Materials and Methods

### mRNA Expression Levels of the STAT Family

We combined the Oncomine and Gene Expression Profiling Interactive Analysis (GEPIA) databases to analyze the expression levels of STAT family members. Oncomine (http://www.oncomine.org) is a comprehensive and user-friendly platform for gene expression, pathway, and network analysis, and it contains 715 datasets with 87,433 samples (Rhodes et al., 2007). The mRNA level of the STAT family in LIHC was explored using Oncomine (*p* < 0.05, fold change (FC) = 2). GEPIA (http://gepia.cancer-pku.cn/) is a network tool that includes LIHC and normal gene expression profiling and interactive analyses based on The Cancer Genome Atlas (TCGA) and Genotype-Tissue Expression (GTEx) data (Tang et al., 2017). The expression of the STAT family in LIHC was obtained using the GEPIA database.

### Protein Expression Levels of the STAT Family

As a comprehensive bioinformatics web resource, the Human Protein Atlas (HPA, https://www.proteinatlas.org) has been designed for mapping all human proteins (Colwill et al., 2011). This database was used to analyze the protein levels of STAT family members in HCC patients.

### Immunoinfiltration Analysis

The Tumor Immune Estimation Resource (TIMER) database is divided into seven modules (https://cistrome.shinyapps.io/timer/), one of which provides a quantitative analysis of immune cell infiltration rates (Li et al., 2017). STAT family members were correlated with the infiltration of six types of immune cells (B cells, CD4^+^ T cells, CD8^+^ T cells, dendritic cells, neutrophils, and macrophages). The TIMER and GEPIA databases were used in combination to comprehensively evaluate the correlations between STAT1 expression and immune checkpoints in HCC. A correlation index value R > 0.1 and *p*-value <0.05 were considered statistically significant in this analysis.

### Chemotherapeutic Response

Computer technology is now widely used in research to find potential therapeutic drugs (Saeed et al., 2020; Alshahrani et al., 2021; Saeed et al., 2021; Tasleem et al., 2021; Zrieq et al., 2021). Genomics of Drug Sensitivity in Cancer (GDSC) was used to predict the chemotherapeutic response of the JAK2 inhibitor lestaurtinib to each sample (Zhao et al., 2020). GDSC (https://www.cancerrxgene.org) is the largest open pharmacogenomics database.

### Cell Lines and Reagents

The human HCC cell line Huh-7 was purchased from the Cell Bank of the Chinese Academy of Sciences. The cells were cultured in DMEM containing 10% FBS, 100 U/mL penicillin, and 100 μg/ml streptomycin. The cells were cultured in a 37°C, 5% CO_2_ incubator. Lestaurtinib was purchased from Tocris (Cat. 3,395, Bristol, United Kingdom).

### MTT Assay

First, the cells were seeded in 96-well plates. After 24 h, the cells were cultured in different concentrations of lestaurtinib (1.0, 0.5, and 0.25 μM), and the results were viewed at 24, 48, or 72 h. MTT was added to each well, and then the cells were incubated in the incubator for 4 h. After that, the culture medium was discarded, 150 μl of DMSO (dimethyl sulfoxide) was added to each well, and the absorbance at 490 nm was measured after shaking. Cell viability (%) = (ODsample−ODblank)/(ODcontrol−ODblank)∗100%.

### Colony Formation Assay

Huh-7 cells were seeded into 24-well plates. After 24 h, the cells were treated with lestaurtinib at concentrations of 0, 0.125, 0.25, 0.50, 1.0, 2.0, and 4.0 μM for three biological replicates and incubated at 37°C for 7 days, with replacement of lestaurtinib every 3 days. The cells were washed with PBS, fixed with cold 100% methanol for 15 min, and then stained with 10% methanol/0.5% crystal violet in 1× PBS for 10 min. Then, brightfield microscopy was performed to quantify the colonies, which were defined as > 50 cells. Colony counts were performed on a total of three representative areas per well according to the cell number parameters mentioned previously. This was performed for each lestaurtinib concentration tested, and the colony counts for each concentration were totaled. Statistical analysis was performed using Prism 7 GraphPad software (Inc., La Jolla, CA, United States). The experimental group was compared with the untreated control group, and *p* < 0.05 indicated statistical significance.

### Transwell Assay

The transwell assay was performed according to a previously reported method (Lu et al., 2019). Huh-7 cells were seeded into the upper layer of transwell chambers (Corning, NY, United States), and cells migrating to the subventricular layer were stained after 48 h of incubation with various concentrations of lestaurtinib (0–1 μM). Migrating cells were observed and photographed using an ECLIPSE Ts2 microscope (Nikon, Japan).

### Quantitative PCR

Total RNA from Huh-7 cells treated with lestaurtinib for 48 h was extracted using TRIzol reagent (Qiagen, United States), and cDNA was synthesized from total RNA (1 μg) using a first-strand synthesis system (Vazyme, China). cDNA was diluted to 2 ng/μl, and then 4 µl was added to 10 µL of 2× FastStart Universal SYBR Green PCR Master Mix (Vazyme, China). Each sample was tested in triplicate using a qPCR system (Applied Biosystems 7,500). Ct values were normalized to the housekeeping gene GAPDH, which was amplified in parallel. The following qPCR primers were used: STAT1: 5′-CGG​CTG​AAT​TTC​GGC​ACC​T-3′ and 5′-CAG​TAA​CGA​TGA​GAG​GAC​CCT-3′; STAT3: 5′-ATC​ACG​CCT​TCT​ACA​GAC​TGC-3′ and 5′-CAT​CCT​GGA​GAT​TCT​CTA​CCA​CT-3′; and GAPDH: 5′-ACT​GCC​ACC​CAG​AAG​ACT-3′ and 5′-GCT​CAG​TGT​AGC​CCA​GGA​T-3′.

### Statistical Analysis

R software (version 3.6.3) and the “rms” package (https://CRAN.R-project.org/package = rms) were applied for the construction of the nomogram. One-way ANOVA was performed using GraphPad 7.0 (Inc., La Jolla, CA, United States), and significant differences were calculated, followed by Student’s t-test or Tukey’s multiple comparison test. *p* < 0.05 indicated a statistically significant difference.

## Results

### Expression of the STAT Family in Hepatocellular Carcinoma

**FIGURE 1:**
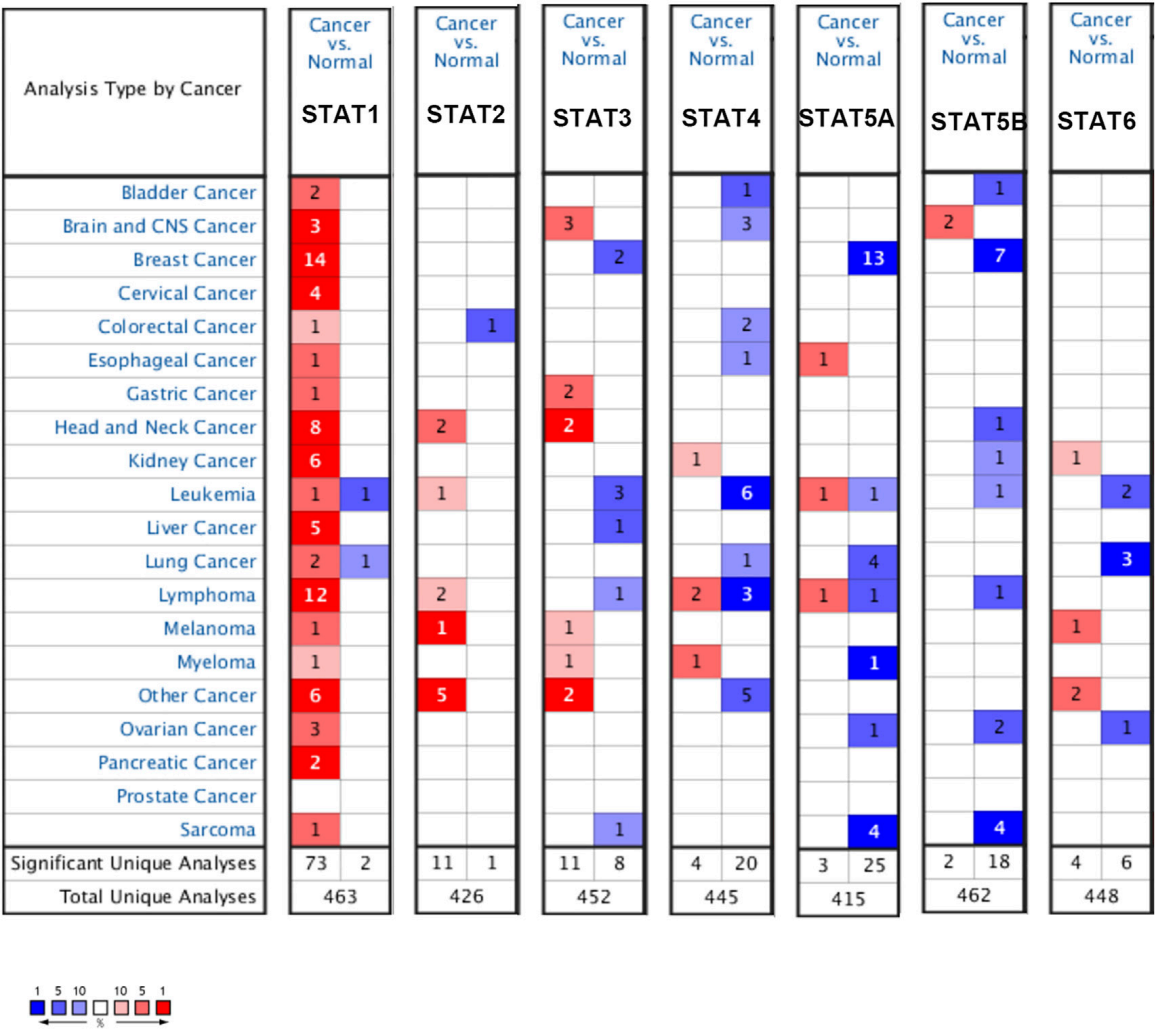
Expression levels of the STAT family in different types of cancers (Oncomine). The panel shows the numbers of datasets with statistically significant mRNA overexpression (red) or downregulated expression (blue) of the STAT family. The threshold was designed with the following parameters: fold change of 1.5 and *p*-value of 0.05.

**FIGURE 2:**
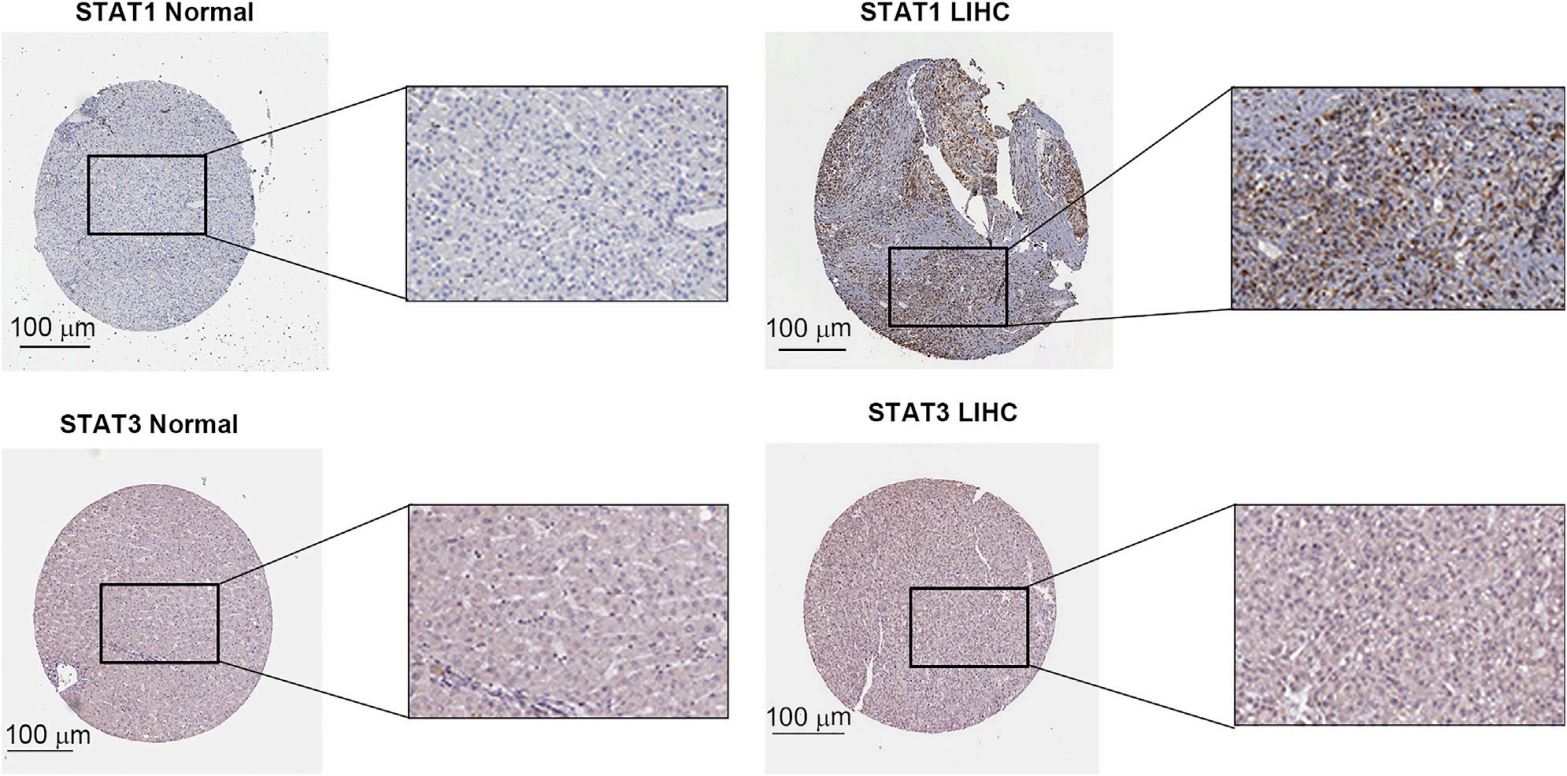
Validation of STAT1 and STAT3 protein levels in The Human Protein Atlas database (HPA).

The expression level of the STAT family in HCC was determined *via* Oncomine, which revealed seven members of the STAT family in humans (Figure 1). Table 1 presents the mRNA levels of STAT family members in HCC patients. The results showed that STAT1 was upregulated in tumor tissues compared with liver tissues. Data obtained by Wurmbach et al. and Mas et al. revealed that STAT1 was upregulated in hepatocellular carcinoma (fold change = 4.016, *p* = 1.23E-6; and fold change = 2.540, *p* = 2.96E-13). Thus, these two datasets indicated that STAT1 was upregulated in HCC. Data obtained by Mas et al. also showed that STAT3 was downregulated in HCC (*p* = 4.66E-9). We also determined the expression levels of STAT family members in HCC using the GEPIA database. When compared with the liver tissue, only the expression of STAT1 was significantly elevated in HCC tissues (Supplementary Figure S1, all *p* < 0.01). The immunohistochemical profiles of the high protein expression of STAT1 (Figure 2), STAT4, STAT5A, STAT5B, and STAT6 in hepatocellular carcinoma tissues were analyzed using the Human Protein Atlas (Supplementary Figure S2).

**TABLE 1:** Datasets of the STAT family in hepatocellular carcinoma (Oncomine).

Gene	Tumor (cases)	Normal (cases)	Fold change	*t*-test	*p*-value	Dataset
STAT1^a^	Hepatocellular carcinoma (Josahkian et al., 2018)	Liver (10)	4.016	5.967	1.23E-6*^,^ ^a^	Wurmbach et al.
	Hepatocellular carcinoma (Lokau et al., 2019)	Liver (19)	2.540	9.395	2.96E-13*^,^ ^a^	Mas et al.
STAT2	Hepatocellular carcinoma (225)	Liver (220)	−1.101	−4.655	1.000	Roessler et al.
STAT3^a^	Hepatocellular carcinoma (Lokau et al., 2019)	Liver (19)	−2.290	−6.908	4.66E-9*^,^ ^a^	Mas et al.
STAT4	Hepatocellular carcinoma (Lokau et al., 2019)	Liver (19)	1.128	1.257	0.108	Mas et al.
STAT5A	Hepatocellular carcinoma (Josahkian et al., 2018)	Liver (10)	−1.078	−1.293	0.896	Wurmbach et al.
STAT5B	Hepatocellular carcinoma (103)	Liver (75)	1.097	1.626	0.053	Chen et al.
STAT6	Hepatocellular carcinoma (103)	Liver (75)	−1.163	−2.314	0.989	Chen et al.

a These results are statistically significant. *p-value <0.001.

### Genetic Alteration of the STAT Family in Hepatocellular Carcinoma

**FIGURE 3:**
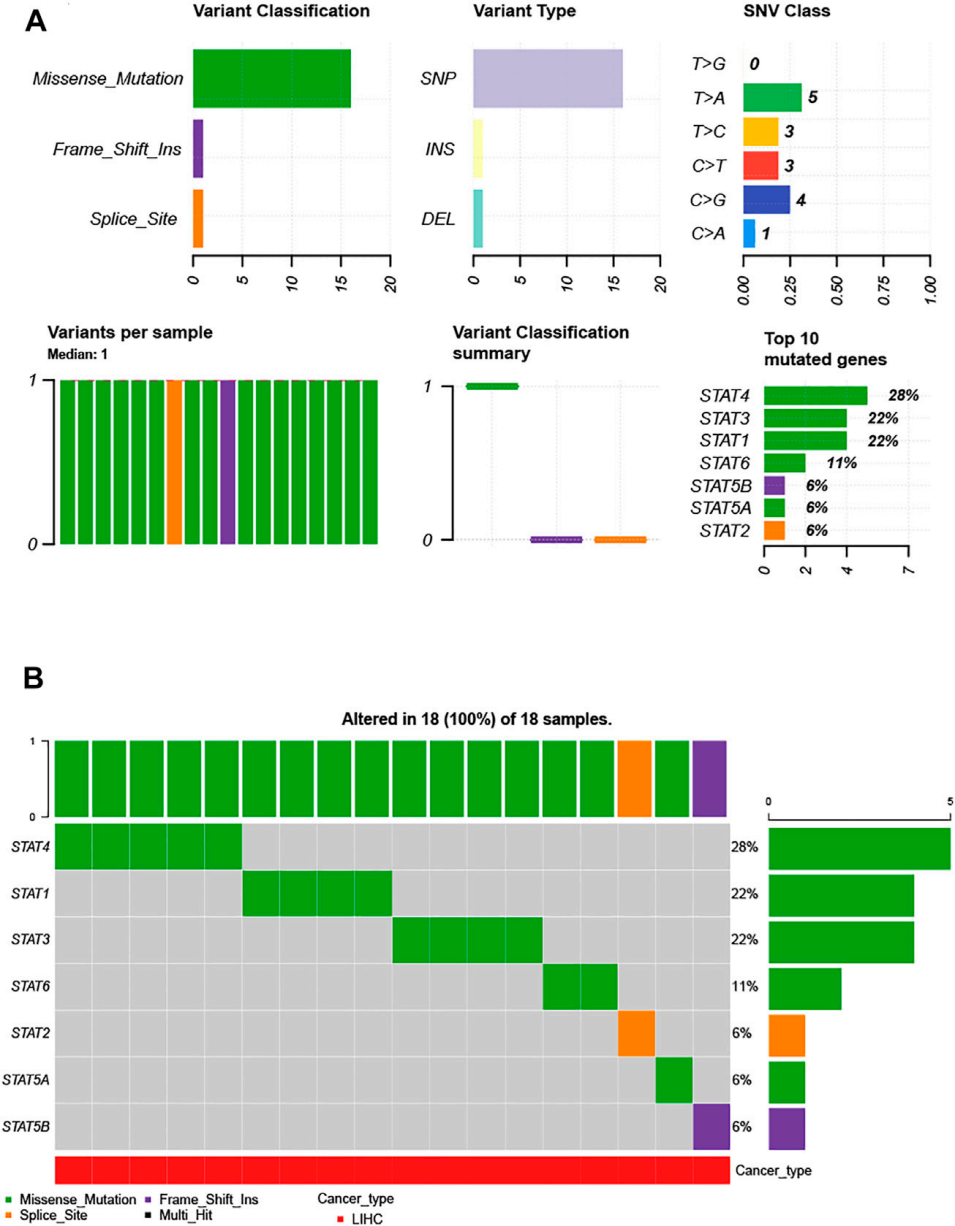
Single-nucleotide variation (SNV) analysis of the STAT family in HCC (GSCALite). **(A)** Summary plot displaying SNV frequency and variant types of the STAT family in HCC, and genetic alterations of the STAT family constitute SNPs, insertions, and deletions. **(B)** Waterfall plot shows the mutation distribution of the STAT family in HCC, and STAT4 (28%), STAT3 (22%), and STAT1 (22%) were the top three most frequently mutated genes among all STAT family members.

Considering the importance of STAT family members in HCC, they are expected to be targets. Therefore, we analyzed the gene mutations of STAT family members. As shown in Figure 3A, genetic alterations in STAT family members involved single-nucleotide polymorphisms (SNPs), insertions, and deletions. The altered form and frequency are shown in Figure 3B. Among all members of the STAT family, STAT4 (28%), STAT1 (22%), and STAT3 (22%) were the top three most frequently mutated genes (Figure 3B). The genetic alteration was a missense mutation (Figure 3B).

### Prognostic Value of the STAT Family in Hepatocellular Carcinoma

**FIGURE 4:**
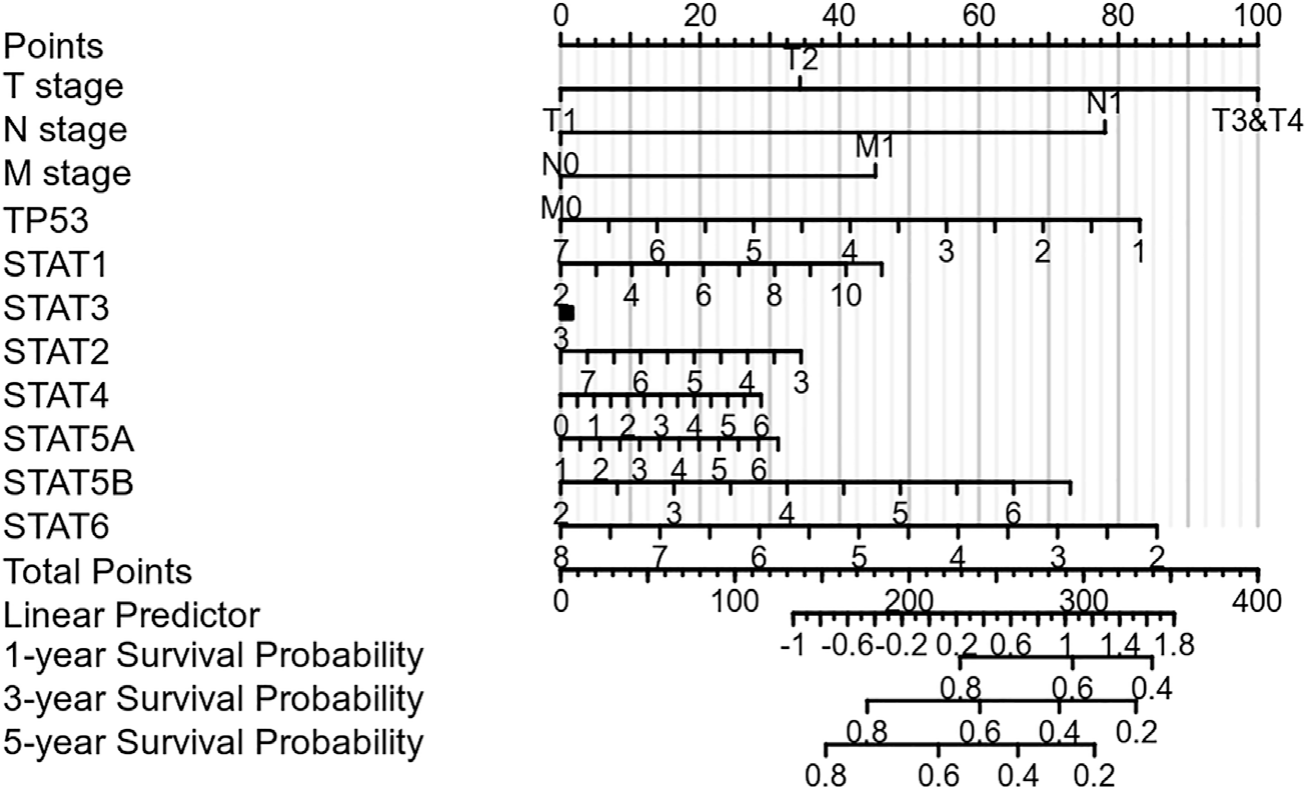
Nomogram integrating the STAT family members and clinical characteristics.

Nomograms were used to accurately predict the 5-year survival rate in patients with hepatocellular carcinoma. This prognostic model integrates STAT family members, TP53, and clinically recognized TNM staging to obtain a comprehensive score that can be used clinically to evaluate the prognosis of HCC. As shown in Figure 4, each item was scored according to the actual situation, and the total score that predicts the survival rate within 5 years can be obtained. The higher the STAT1 expression, the higher the corresponding individual scores, the higher the total score, and the worse the prognosis of that patient. Figure 4 shows that STAT1 is not a protective factor for patients with HCC.

### Sensitivity of the JAK2 Inhibitor Lestaurtinib to Hepatocellular Carcinoma

**FIGURE 5:**
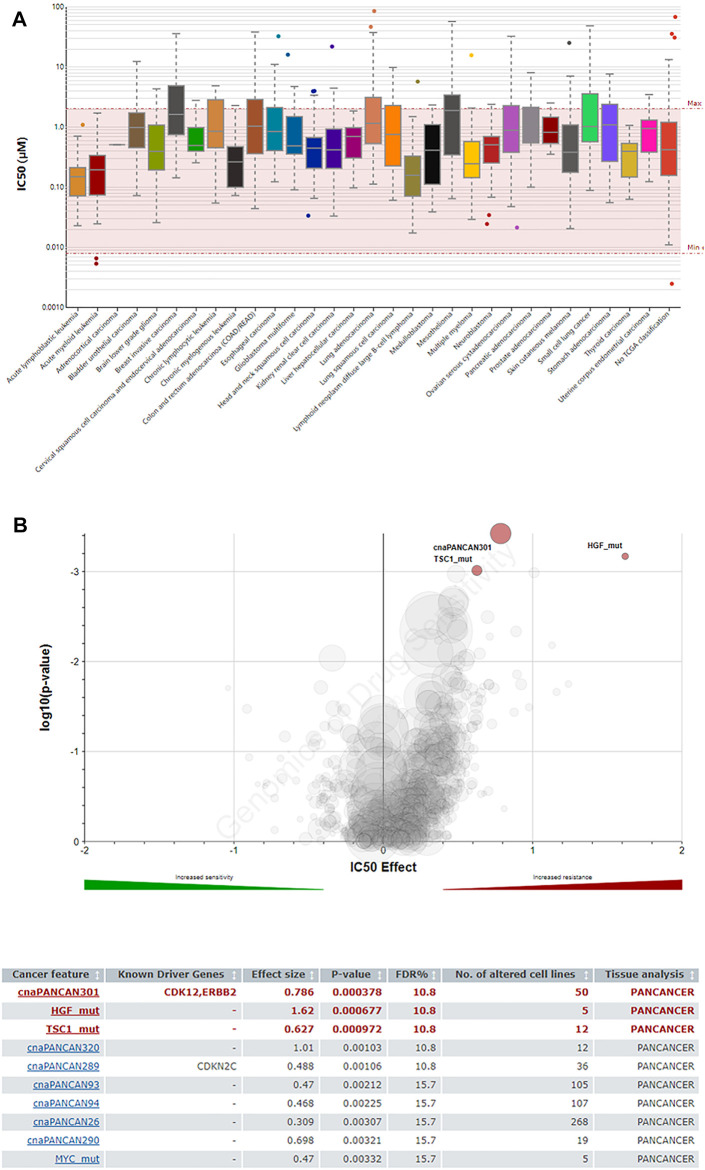
Predictive effects of the JAK2 inhibitor lestaurtinib on cancer cells (GDSC). **(A)** IC_50_ distribution of lestaurtinib by tissue type. **(B)** Volcano plot of the sensitivity of cancer cell lines to lestaurtinib.

JAK2 is the upstream regulator of STAT family members. In recent years, drug research and development for JAK have become relatively mature. Therefore, JAK was selected as a feasible target to inhibit the downstream effect of the JAK/STAT pathway. Lestaurtinib, a multikinase inhibitor, can specifically block JAK2 (Pinto et al., 2018). However, due to the off-target effect of kinase inhibitors, the actual anticancer mechanism is not clear (Tullemans et al., 2018; Stathopoulou et al., 2019). The GDSC database was used to predict the IC_50_ value of lestaurtinib and the response sensitivity of lestaurtinib to liver cancer cell lines (Figure 5A). In Figure 5B, HGF and TSC1 mutant hepatocytes were more sensitive to lestaurtinib.

### Effects of the JAK2 Inhibitor Lestaurtinib on Liver Cancer Cells

**FIGURE 6:**
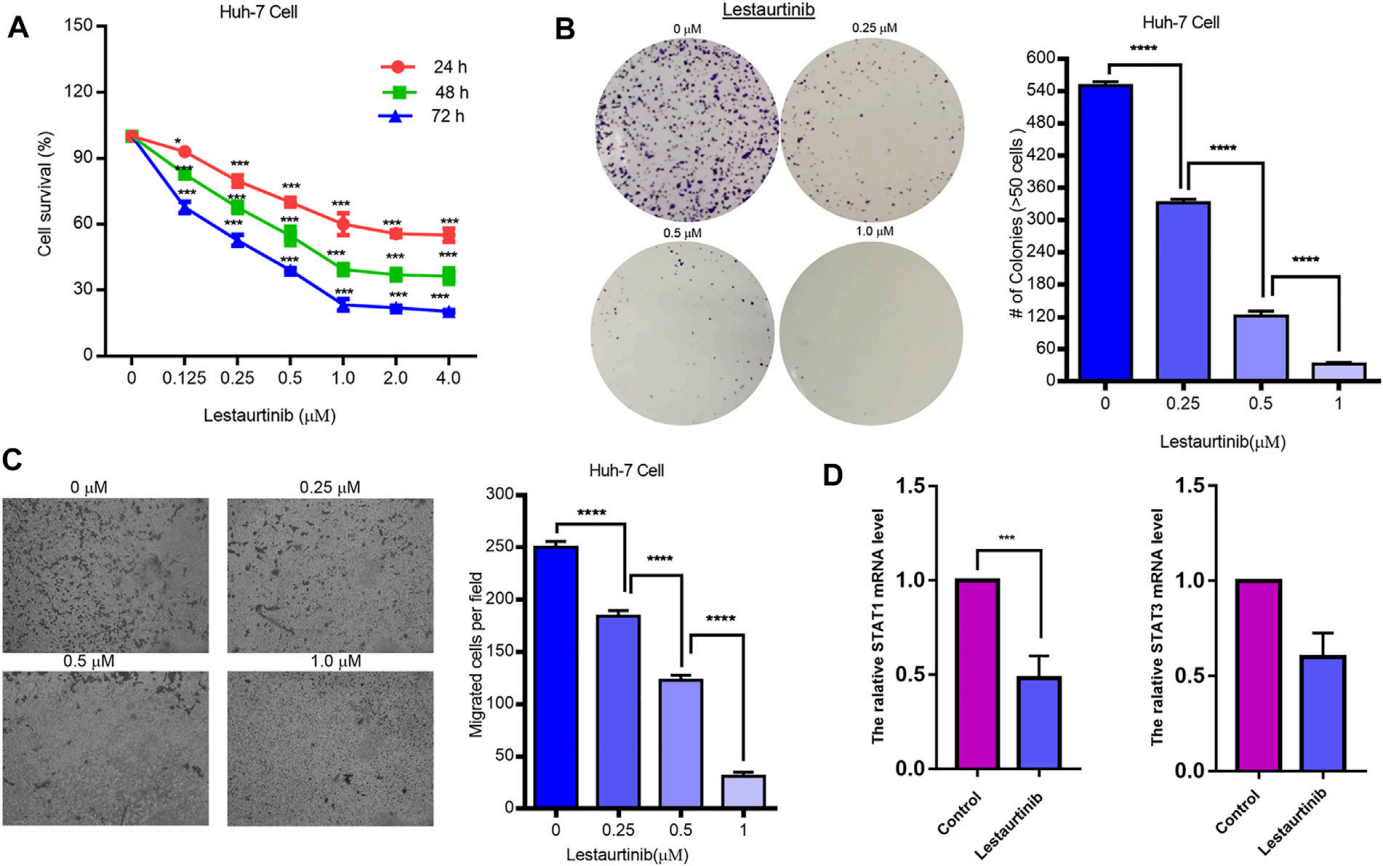
Effects of the JAK2 inhibitor lestaurtinib on liver cancer cells. **(A)** MTT results of lestaurtinib in Huh-7 cells. **(B)** Colony formation and **(C)** transwell migration results of lestaurtinib on Huh-7 cells. **(D)** mRNA expression of STAT1 and STAT3. Data were obtained from three independent experiments. *p < 0.05, **p < 0.01, and ***p < 0.001, compared with the control group.

As shown in Figure 6A, lestaurtinib attenuated regenerative Huh-7 cells in a time- and dose-dependent manner after 72 h of treatment. The IC_50_s of lestaurtinib in Huh-7 cells were >4 μM for 24 h, 0.67 ± 0.17 μM for 48 h, and 0.25 ± 0.13 μM. After 72 h of treatment with lestaurtinib, the drug successfully delayed or inhibited the colony formation of Huh-7 cells (Figure 6B). In addition, we investigated the effect of lestaurtinib on Huh-7-cell migration. As shown in Figure 6C, lestaurtinib significantly inhibited the migration of Huh-7 cells. The aforementioned experimental results show that lestaurtinib can inhibit the proliferation of hepatoma cells in a time- and dose-dependent manner and can also impair the migration ability of hepatoma cells. As shown in Figure 6D, our experimental results suggest that lestaurtinib caused an inhibition of STAT1 expression.

### Correlation Between STAT Family Expression and Immune Infiltration Level in Hepatocellular Carcinoma

**FIGURE 7:**
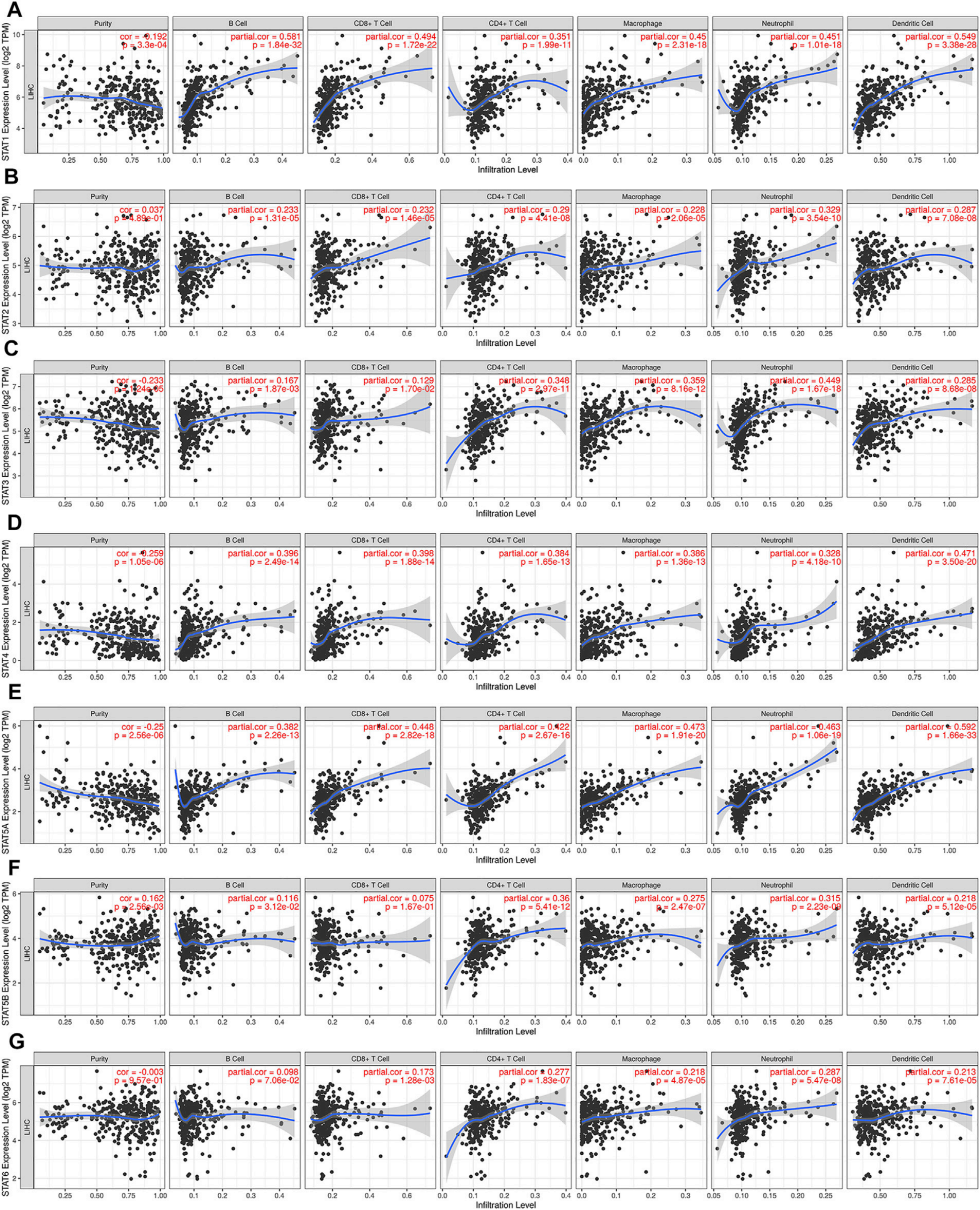
Relationship between STAT family expression levels and immune infiltration levels in HCC (TIMER). Correlation between the abundance of immune cells and the expression of **(A)** STAT1, **(B)** STAT2, **(C)** STAT3, **(D)** STAT4, **(E)** STAT5A, **(F)** STAT5B, and **(G)** STAT6 in HCC.

The relationship between the expression of STAT family members and level of immune infiltrating cells in patients with liver cancer was evaluated using TIMER online analysis. The analysis results are shown in Figure 7. We found that compared with other members of the STAT family, STAT1 attracted our attention, and the correlations between STAT1 and infiltrating immune cells are as follows: B cells (r = 0.581); CD8^+^ T cells (r = 0.494); CD4^+^ T cells (r = 0.351); macrophages (r = 0.45); neutrophils (r = 0.451); and dendritic cells (r = 0.549). STAT1 was positively correlated with the infiltration of B cells, CD8^+^ T cells, CD4^+^ T cells, macrophages, neutrophils, and dendritic cells in HCC patients, and the correlation value was greater than 0.3 (Figure 7A). STAT1 has the strongest correlation with immune infiltrating cells, regulating the immune microenvironment, and thus affects the clinical outcome of patients with liver cancer.

### Correlation Between STAT1 Levels and Immune Cell Biomarkers in Hepatocellular Carcinoma

To further explore the role of STAT1 in tumor immunity, we used the GEPIA database to determine the correlation between STAT1 and the expression of immune cell biomarkers in HCC. STAT1 and B-cell biomarkers (CD19 and CD79A), CD8^+^ T-cell biomarkers (CD8A and CD8B), CD4^+^ T-cell biomarkers (CD4), M1 macrophage biomarkers (NOS2, IRF5, and PTGS2), M2 macrophage biomarkers (CD163, VSIG4, and MS4A4A), neutrophil biomarkers (ITGAM and CCR7), and dendritic cell biomarkers in HCC (HLA-DPB1, HLA-DQB1, HLA-DRA, HLA-DPA1, CD1C, NRP1, and ITGAX) are shown in Table 2. The positive correlation between STAT1 and immune cell infiltration was partially supported by these findings.

**TABLE 2:** Correlation analysis between STAT1 and biomarkers of immune cells in HCC (GEPIA).

Immune cell	Biomarker	R-value	*p*-value
B-cell	CD19	0.38^a^	7.3e-14***^,^ ^a^
	CD79A	0.47^a^	6.8e-22***^,^ ^a^
CD8^+^ T-cell	CD8A	0.54^a^	4.4e-29***^,^ ^a^
	CD8B	0.45^a^	4.7e-20***^,^ ^a^
CD4^+^ T-cell	CD4	0.45^a^	5.3e-20***^,^ ^a^
M1 macrophage	NOS2	0.16^a^	0.0015**^,^ ^a^
	IRF5	0.31^a^	7.5e-10***^,^ ^a^
	PTGS2	0.4^a^	1e-15***^,^ ^a^
M2 macrophage	CD163	0.34^a^	1.4e-11***^,^ ^a^
	VSIG4	0.39^a^	8.5e-15***^,^ ^a^
	MS4A4A	0.47^a^	2.1e-21***^,^ ^a^
	CD68	0.43^a^	9.5e-18***^,^ ^a^
Neutrophil	CEACAM8	0.047	0.37
	ITGAM	0.42^a^	6.7e-17***^,^ ^a^
	CCR7	0.47^a^	2e-21***^,^ ^a^
Dendritic cell	HLA-DPB1	0.56^a^	1.9e-31***
	HLA-DQB1	0.31^a^	1.3e-09***^,^ ^a^
	HLA-DRA	0.58^a^	3.2e-34***^,^ ^a^
	HLA-DPA1	0.57^a^	2.1e-33***^,^ ^a^
	CD1C	0.42^a^	1.6e-17***^,^ ^a^
	NRP1	0.37^a^	2.3e-13***^,^ ^a^
	ITGAX	0.51^a^	2.8e-26***^,^ ^a^

a These results are statistically significant; *p-value <0.05; **p-value <0.01; ***p-value <0.001.

### Correlation Between STAT1 Levels and Immune Checkpoints in Hepatocellular Carcinoma

**FIGURE 8:**
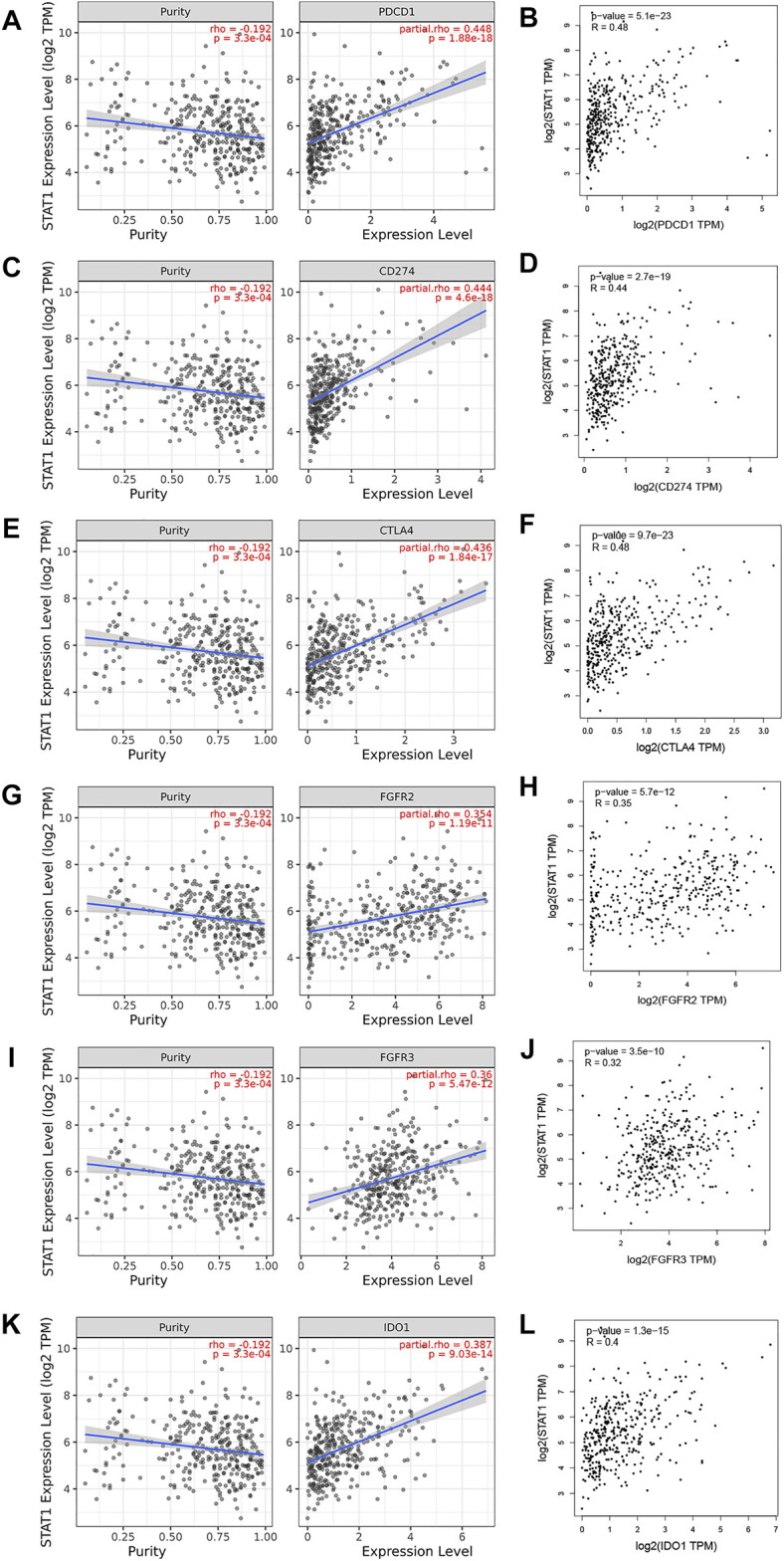
Correlation of STAT1 expression with immune checkpoint expression in HCC (TIMER and GEPIA). The expression correlation of STAT1 with **(A,B)** PD1, **(C,D)** PD-L1, **(E,F)** CTLA-4, **(G,H)** FGFR2, **(I,J)** FGFR3, and **(K,L)** IDO1 in HCC.

Based on the aforementioned analysis, STAT1 acts as a tumor-promoting factor in hepatocellular carcinoma and can be used to evaluate the prognosis of HCC patients. Typical and important immunological checkpoints are responsible for tumor immune escape. Therefore, this study further evaluated the correlation between STAT1 and immune checkpoints (PD1, PD-L1, CTLA-4, FGFR2, FGFR3, and IDO1). The results are shown in Figure 8, and the TIMER analysis showed that STAT1 was significantly positively correlated with PD1 (r = 0.448); PD-L1 (r = 0.444); CTLA-4 (r = 0.436); FGFR2 (r = 0.354); FGFR3 (r = 0.36); and IDO1 (r = 0.387) in HCC, and these checkpoints were regulated by purity. The results were consistent with those from the GEPIA database analysis and showed that STAT1 was also significantly positively correlated with PD1 (r = 0.48); PD-L1 (r = 0.44); CTLA-4 (r = 0.48); FGFR2 (r = 0.35); FGFR3 (r = 0.32); and IDO1 (r = 0.4) in HCC. Based on the aforementioned results, we can reasonably infer that tumor immune escape may be involved in the STAT1-mediated carcinogenesis of HCC.

## Discussion

HCC is a cancer with multiple etiologies and widespread prevalence. Because of the lack of typical clinical manifestations and a high postoperative recurrence rate in HCC patients, only a few subjects can receive potentially effective treatment (Chen et al., 2017; Federico et al., 2020). Largely refractory to current treatments, HCC is the third leading cause of cancer-related deaths worldwide (Bray et al., 2020). Therefore, this study combined bioinformatics and pharmacological experiments to explore new therapeutic targets to improve the prognosis of hepatocellular carcinoma patients.

The STAT family has now been shown to affect fundamental cellular mechanisms, including cell proliferation, invasion, apoptosis, and cellular immunity, by modulating cytokine signaling (Verhoeven et al., 2020). The JAK/STAT signaling pathway was found to be associated with the genesis and progression of tumors, such as breast cancer, prostate cancer, bladder cancer, and lung cancer (Groner and von Manstein, 2017; Yang X. et al., 2019; Huang et al., 2020). To our knowledge, the specific expression and associated roles of STAT family members in HCC have not been elucidated. Therefore, the current bioinformatics analysis aimed to evaluate the expression level, prognostic value, and mutation status of the STAT family in HCC patients to screen candidate therapeutic targets for further pharmacological experiments.

STAT1, STAT2, STAT3, STAT4, STAT5A, STAT5B, and STAT6 are included in the mammalian family of STATs, and they are all encoded by distinct genes. In our study, the differential expression analysis results showed that among all STAT family members, only STAT1 was upregulated in HCC compared with normal tissues at both the mRNA and protein levels. Previous studies suggested that some STAT family members may serve as biomarkers for various types of cancer. Juliana et al. suggested that STAT1 functioned as both a prognostic and predictive biomarker in ovarian cancer (Josahkian et al., 2018), and Guo et al. revealed that STAT5A serves as an immune checkpoint inhibitor and biomarker for the diagnosis and prognosis of liver hepatocellular carcinoma (Guo et al., 2020).

We next performed genetic alteration and immune infiltration analyses of the STAT family in HCC and found that STAT4 (28%), STAT1 (22%), and STAT3 (22%) were the top three most frequently mutated genes, with missense mutations being the most common genetic alteration type. Gene mutations are closely related to the pathogenesis and progression of HCC and seriously affect the prognosis of HCC patients.

A nomogram integrating the clinical characteristics of HCC patients was constructed, and the scoring items included STAT family members, TP53, and TNM stages. In this constructed nomogram, STAT6 was the highest weighted score among seven members of the STAT family, followed by STAT5B and STAT1. This qualifies STAT1 as a novel marker for evaluating the prognosis of patients with hepatocellular carcinoma.

Current research suggests that STAT1 and STAT3 are nuclear transcription factors that regulate genes involved in the cell cycle, cell survival, and immune response (Butturini et al., 2020). Our preliminary studies were consistent with these conclusions. STAT1 expression was positively correlated with the infiltration of B cells, CD8^+^ T cells, CD4^+^ T cells, macrophages, neutrophils, and dendritic cells. Lokau Juliane et al. suggested that the JAK/STAT cascade is critical for HCC development (Lokau et al., 2019). In another study, downregulating the level of miR-196a or miR-196b regulated the JAK/STAT pathway by targeting SOCS2 to inhibit the occurrence and development of HCC, thus providing a new potential target for the prognosis and treatment of HCC (Ren et al., 2019). Therefore, STAT family members may be important regulators affecting the occurrence and progression of HCC. STAT1, as a part of the JAK/STAT signaling cascade, mainly mediates various types of interferon (IFN) reactions. STAT1 regulation involves a variety of biological functions, such as antibacterial activity, cell proliferation, and cell death, and plays an important role in the innate and adaptive arms of the immune system (Meissl et al., 2017). It is brought to our attention that STAT1 is usually considered a tumor suppressor, while increasing evidence suggests that it can also act as a tumor promoter. Buyun Ma et al. studied the function of nonphosphorylated (U-) STAT1 in liver cancer. They found that (U-) STAT1 was significantly upregulated in HCC tumor tissues and mainly expressed in the cytoplasm while the deletion of (U-) STAT1 in hepatoma cells may block the cell cycle and limit cell growth (Ma et al., 2019). Given the above, STAT1 was selected for further studies.

A large number of studies have confirmed that the efficacy and prognosis of chemotherapy, radiotherapy, or immunotherapy in patients with hepatocellular carcinoma are closely related to the tumor-infiltrating microenvironment (Zhang et al., 2018; Lyu et al., 2020). The tumor microenvironment is infiltrated with a variety of immune cells, mainly B cells, CD8^+^ T cells, CD4^+^ T cells, dendritic cells, macrophages, and neutrophils. Our work showed that STAT1 was significantly positively correlated with the aforementioned infiltrating immune cells in HCC. Furthermore, STAT1 was also significantly positively correlated with these infiltrating immune cells.

Patient prognosis depends not only on a tumor microenvironment that presents immune cell infiltration but also on immune checkpoints (Chae et al., 2018). Therefore, we also evaluated the relationship between STAT1 and immune checkpoints. STAT1 was significantly positively correlated with PD1 (r = 0.448), PD-L1 (r = 0.444), CTLA-4 (r = 0.436), FGFR2 (r = 0.354), FGFR3 (r = 0.36), and IDO1 (r = 0.387) in HCC, suggesting that targeting STAT1 may improve the efficacy of immunotherapy for hepatocellular carcinoma. These findings suggest that it may be involved in tumor immune escape, partially explaining the oncogenic role of STAT1 in HCC.

**FIGURE 9:**
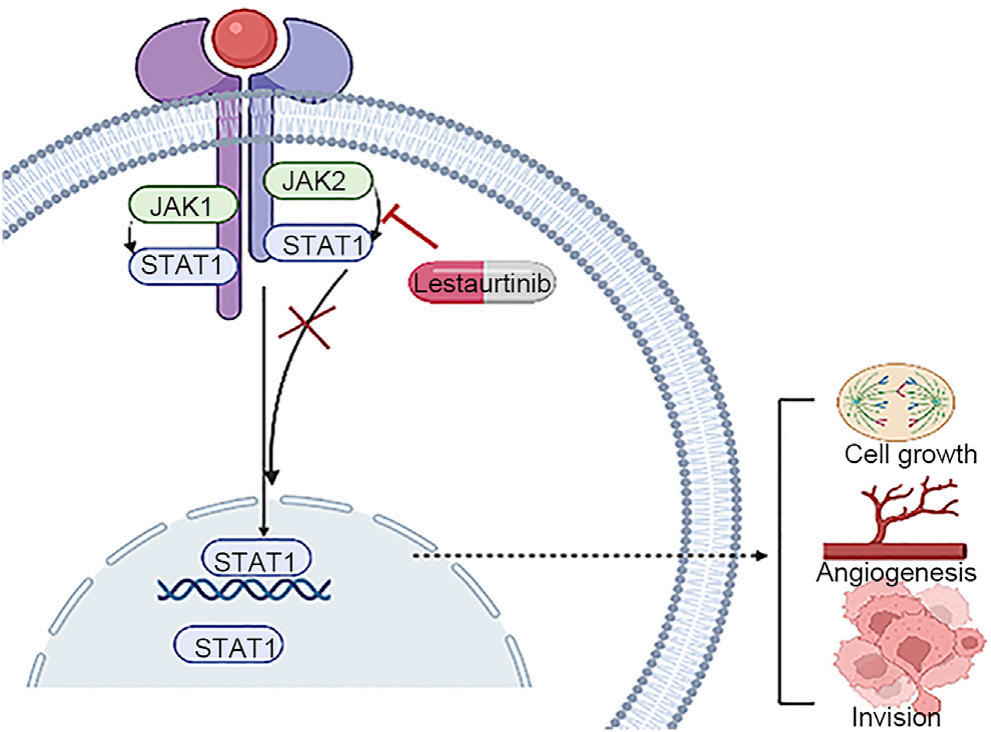
Lestaurtinib has the potential to inhibit the proliferation of hepatocellular carcinoma.

During the occurrence and development of HCC, JAK/STAT signaling is abnormally activated, resulting in the imbalance of downstream target genes involved in the control of survival, angiogenesis, stem cells, immune monitoring, invasion, and metastasis. The JAK/STAT pathway is a promising target for the treatment of hepatocellular carcinoma (Hin Tang et al., 2020). Lestaurtinib is a multikinase inhibitor, and JAK2 is its target (Shabbir and Stuart, 2010). Extensive and mature *in vitro* studies on lestaurtinib have demonstrated its highly positive safety record and oral bioavailability (Knapper et al., 2017). However, due to the existence of off-target effects (Munoz, 2017), the real anticancer mechanism is not clear. Lestaurtinib has previously been used in several clinical trials of hematogenous malignancies, although its potential utility for liver cancer has not been explored (Diaz et al., 2011; Köhler et al., 2012). In this study, we performed *in vitro* experiments using lestaurtinib as a treatment for patients with liver cancer and verified that lestaurtinib, a tyrosine kinase inhibitor, can inhibit the growth of liver cancer cells (Figure 9).

In conclusion, the goal of our study was to determine whether STAT1 can be used as a candidate factor for further research based on its role as a biomarker and therapeutic target in HCC. In addition, we used the GDSC database to analyze the targets of the JAK/STAT pathway for drug screening. The JAK inhibitor lestaurtinib was very sensitive to liver cancer cell lines, and its effects on the viability of liver cancer cells were examined by pharmacological experiments. However, our study had several limitations. We will explore the effects of lestaurtinib in hepatocellular carcinoma through *in vitro* and *in vivo* systemic studies in order to provide a new strategy for the treatment of hepatocellular carcinoma. The *in vivo* studies focus on addressing how to limit toxicity at optimal biological doses.

## Data Availability

The original contributions presented in the study are included in the article/Supplementary Material, further inquiries can be directed to the corresponding author.

## References

[B1] Alshahrani M. Y. Alshahrani K. M. Tasleem M. Akeel A. Almeleebia T. M. Ahmad I. (2021). Computational Screening of Natural Compounds for Identification of Potential Anti-cancer Agents Targeting MCM7 Protein. Molecules (Basel, Switzerland) 26 (19). 10.3390/molecules26195878 PMC8510405 34641424

[B3] Banerjee S. Biehl A. Gadina M. Hasni S. Schwartz D. M. (2017). JAK-STAT Signaling as a Target for Inflammatory and Autoimmune Diseases: Current and Future Prospects. Drugs 77 (5), 521–546. 10.1007/s40265-017-0701-9 28255960 PMC7102286

[B2] Bray F. Ferlay J. Soerjomataram I. Siegel R. Torre L. Jemal A. (2020). Erratum: Global Cancer Statistics 2018: GLOBOCAN Estimates of Incidence and Mortality Worldwide for 36 Cancers in 185 Countries. CA Cancer J. Clin. 70 (4), 313. 10.3322/caac.21609 30207593

[B4] Butturini E. Carcereri de Prati A. Mariotto S. (2020). Redox Regulation of STAT1 and STAT3 Signaling. Int. J. Mol. Sci. 21 (19). 10.3390/ijms21197034 PMC7582491 32987855

[B5] Chae Y. K. Arya A. Iams W. Cruz M. R. Chandra S. Choi J. (2018). Current Landscape and Future of Dual Anti-CTLA4 and PD-1/pd-L1 Blockade Immunotherapy in Cancer; Lessons Learned from Clinical Trials with Melanoma and Non-small Cell Lung Cancer (NSCLC). J. Immunotherapy Cancer 6 (1), 39. 10.1186/s40425-018-0349-3 PMC5956851 29769148

[B6] Chen H.-Y. Chen Y.-M. Wu J. Yang F.-C. Lv Z. Qian Y.-G. (2017). Effects of HGF Gene Polymorphisms and Protein Expression on Transhepatic Arterial Chemotherapeutic Embolism Efficacy and Prognosis in Patients with Primary Liver Cancer. Ott Vol. 10, 803–810. 10.2147/ott.s115035 PMC5315352 28243116

[B7] Colwill K. Gräslund S. Gräslund S. (2011). A Roadmap to Generate Renewable Protein Binders to the Human Proteome. Nat. Methods 8 (7), 551–558. 10.1038/nmeth.1607 21572409

[B8] Diaz T. Navarro A. Ferrer G. Gel B. Gaya A. Artells R. (2011). Lestaurtinib Inhibition of the Jak/STAT Signaling Pathway in Hodgkin Lymphoma Inhibits Proliferation and Induces Apoptosis. PLoS One 6 (4), e18856. 10.1371/journal.pone.0018856 21533094 PMC3080386

[B9] Federico P. Petrillo A. Giordano P. Bosso D. Fabbrocini A. Ottaviano M. (2020). Immune Checkpoint Inhibitors in Hepatocellular Carcinoma: Current Status and Novel Perspectives. Cancers (Basel) 12 (10). 10.3390/cancers12103025 PMC7603151 33080958

[B10] Groner B. von Manstein V. (2017). Jak Stat Signaling and Cancer: Opportunities, Benefits and Side Effects of Targeted Inhibition. Mol. Cell. Endocrinol. 451, 1–14. 10.1016/j.mce.2017.05.033 28576744

[B11] Guo L. Fang T. Jiang Y. Liu D. (2020). Identification of Immune Checkpoint Inhibitors and Biomarkers Among STAT Family in Stomach Adenocarcinoma. Am. J. Transl Res. 12 (9), 4977–4997. 33042401 PMC7540141

[B12] Hin Tang J. J. J. J. Hao Thng D. K. Lim J. J. Toh T. B. (2020). JAK/STAT Signaling in Hepatocellular Carcinoma. Hepat. Oncol. 7 (1), Hep18. 10.2217/hep-2020-0001 32273976 PMC7137178

[B13] Huang W. Li Y. Zhang C. Zha H. Zhou X. Fu B. (2020). IGF2BP3 Facilitates Cell Proliferation and Tumorigenesis via Modulation of JAK/STAT Signalling Pathway in Human Bladder Cancer. J. Cel. Mol. Med. 24 (23), 13949–13960. 10.1111/jcmm.16003 PMC7753985 33094561

[B14] Josahkian J. A. Saggioro F. P. Vidotto T. Ventura H. T. Candido Dos Reis F. J. de Sousa C. B. (2018). Increased STAT1 Expression in High Grade Serous Ovarian Cancer Is Associated with a Better Outcome. Int. J. Gynecol. Cancer 28 (3), 459–465. 10.1097/igc.0000000000001193 29303938

[B15] Kim S. K. Takeda H. Takai A. Matsumoto T. Kakiuchi N. Yokoyama A. (2019). Comprehensive Analysis of Genetic Aberrations Linked to Tumorigenesis in Regenerative Nodules of Liver Cirrhosis. J. Gastroenterol. 54 (7), 628–640. 10.1007/s00535-019-01555-z 30756187

[B16] Knapper S. Russell N. Gilkes A. Hills R. K. Gale R. E. Cavenagh J. D. (2017). A Randomized Assessment of Adding the Kinase Inhibitor Lestaurtinib to First-Line Chemotherapy for FLT3-Mutated AML. Blood 129 (9), 1143–1154. 10.1182/blood-2016-07-730648 27872058 PMC5364440

[B17] Köhler J. Erlenkamp G. Eberlin A. Rumpf T. Slynko I. Metzger E. (2012). Lestaurtinib Inhibits Histone Phosphorylation and Androgen-dependent Gene Expression in Prostate Cancer Cells. PLoS One 7 (4), e34973. 10.1371/journal.pone.0034973 22532837 PMC3332061

[B18] Li T. Fan J. Wang B. Traugh N. Chen Q. Liu J. S. (2017). TIMER: A Web Server for Comprehensive Analysis of Tumor-Infiltrating Immune Cells. Cancer Res. 77 (21), e108–e110. 10.1158/0008-5472.can-17-0307 29092952 PMC6042652

[B19] Llovet J. M. Kelley R. K. Villanueva A. Singal A. G. Pikarsky E. Roayaie S. (2021). Hepatocellular Carcinoma. Nat. Rev. Dis. Primers 7 (1), 6. 10.1038/s41572-020-00240-3 33479224 PMC13537433

[B20] Lokau J. Schoeder V. Haybaeck J. Garbers C. (2019). Jak-Stat Signaling Induced by Interleukin-6 Family Cytokines in Hepatocellular Carcinoma. Cancers (Basel) 11 (11). 10.3390/cancers11111704 PMC6896168 31683891

[B21] Lu H.-Y. Zu Y.-X. Jiang X.-W. Sun X.-T. Liu T.-Y. Li R.-L. (2019). Novel ADAM-17 Inhibitor ZLDI-8 Inhibits the Proliferation and Metastasis of Chemo-Resistant Non-small-cell Lung Cancer by Reversing Notch and Epithelial Mesenchymal Transition *In Vitro* and *In Vivo* . Pharmacol. Res. 148, 104406. 10.1016/j.phrs.2019.104406 31442576

[B22] Lyu L. Yao J. Wang M. Zheng Y. Xu P. Wang S. (2020). Overexpressed Pseudogene HLA-DPB2 Promotes Tumor Immune Infiltrates by Regulating HLA-DPB1 and Indicates a Better Prognosis in Breast Cancer. Front. Oncol. 10, 1245. 10.3389/fonc.2020.01245 32903535 PMC7438735

[B23] Ma B. Chen K. Liu P. Li M. Liu J. Sideras K. (2019). Dichotomal Functions of Phosphorylated and Unphosphorylated STAT1 in Hepatocellular Carcinoma. J. Mol. Med. 97 (1), 77–88. 10.1007/s00109-018-1717-7 30456450 PMC6326978

[B24] Mak L.-Y. Cruz-Ramón V. Chinchilla-López P. Torres H. A. LoConte N. K. Rice J. P. (2018). Global Epidemiology, Prevention, and Management of Hepatocellular Carcinoma. Am. Soc. Clin. Oncol. Educ. Book 38, 262–279. 10.1200/edbk_200939 30231359

[B25] Meissl K. Macho-Maschler S. Müller M. Strobl B. (2017). The Good and the Bad Faces of STAT1 in Solid Tumours. Cytokine 89, 12–20. 10.1016/j.cyto.2015.11.011 26631912

[B26] Mohanty S. K. Yagiz K. Pradhan D. Luthringer D. J. Amin M. B. Alkan S. (2017). STAT3 and STAT5A Are Potential Therapeutic Targets in Castration-Resistant Prostate Cancer. Oncotarget 8 (49), 85997–86010. 10.18632/oncotarget.20844 29156772 PMC5689662

[B27] Munoz L. (2017). Non-kinase Targets of Protein Kinase Inhibitors. Nat. Rev. Drug Discov. 16 (6), 424–440. 10.1038/nrd.2016.266 28280261

[B28] Owen K. L. Brockwell N. K. Parker B. S. (2019). JAK-STAT Signaling: A Double-Edged Sword of Immune Regulation and Cancer Progression. Cancers (Basel) 11 (12). 10.3390/cancers11122002 PMC6966445 31842362

[B29] Pastuszak-Lewandoska D. Domańska-Senderowska D. Kordiak J. Antczak A. Czarnecka K. H. Migdalska-Sęk M. (2017). Immunoexpression Analysis of Selected JAK/STAT Pathway Molecules in Patients with Non- Small-Cell Lung Cancer. Pol. Arch. Intern. Med. 127 (11), 758–764. 10.20452/pamw.4115 28972958

[B30] Pinto N. Prokopec S. D. Vizeacoumar F. Searle K. Lowerison M. Ruicci K. M. (2018). Lestaurtinib Is a Potent Inhibitor of Anaplastic Thyroid Cancer Cell Line Models. PLoS One 13 (11), e0207152. 10.1371/journal.pone.0207152 30419054 PMC6231667

[B31] Ren W. Wu S. Wu Y. Liu T. Zhao X. Li Y. (2019). MicroRNA-196a/-196b Regulate the Progression of Hepatocellular Carcinoma through Modulating the JAK/STAT Pathway via Targeting SOCS2. Cell Death Dis 10 (5), 333. 10.1038/s41419-019-1530-4 30988277 PMC6465376

[B32] Rhodes D. R. Kalyana-Sundaram S. Mahavisno V. Varambally R. Yu J. Briggs B. B. (2007). Oncomine 3.0: Genes, Pathways, and Networks in a Collection of 18,000 Cancer Gene Expression Profiles. Neoplasia 9 (2), 166–180. 10.1593/neo.07112 17356713 PMC1813932

[B33] Saeed M. Saeed A. Alam M. J. Alreshidi M. (2020). Identification of Persuasive Antiviral Natural Compounds for COVID-19 by Targeting Endoribonuclease NSP15: A Structural-Bioinformatics Approach. Molecules 25 (23). 10.3390/molecules25235657 PMC7729992 33271751

[B34] Saeed M. Shoaib A. Tasleem M. Alabdallah N. M. Alam M. J. Asmar Z. E. (2021). Assessment of Antidiabetic Activity of the Shikonin by Allosteric Inhibition of Protein-Tyrosine Phosphatase 1B (PTP1B) Using State of Art: An In Silico and *In Vitro* Tactics. Molecules (Basel, Switzerland) 26 (13). 10.3390/molecules26133996 PMC8271486 34208908

[B35] Shabbir M. Stuart R. (2010). Lestaurtinib, a Multitargeted Tyrosinse Kinase Inhibitor: from Bench to Bedside. Expert Opin. Investig. Drugs 19 (3), 427–436. 10.1517/13543781003598862 20141349

[B36] Sia D. Villanueva A. Friedman S. L. Llovet J. M. (2017). Liver Cancer Cell of Origin, Molecular Class, and Effects on Patient Prognosis. Gastroenterology 152 (4), 745–761. 10.1053/j.gastro.2016.11.048 28043904 PMC12160040

[B37] Stathopoulou K. Schobesberger S. Bork N. I. Sprenger J. U. Perera R. K. Sotoud H. (2019). Divergent Off-Target Effects of RSK N-Terminal and C-Terminal Kinase Inhibitors in Cardiac Myocytes. Cell Signal. 63, 109362. 10.1016/j.cellsig.2019.109362 31344438

[B38] Sung H. Ferlay J. Siegel R. Laversanne M. Soerjomataram I. Jemal A. (2021). Global Cancer Statistics 2020: GLOBOCAN Estimates of Incidence and Mortality Worldwide for 36 Cancers in 185 Countries. CA: a Cancer J. clinicians 71 (3), 209–249. 10.3322/caac.21660 33538338

[B39] Tang Z. Li C. Kang B. Gao G. Li C. Zhang Z. (2017). GEPIA: a Web Server for Cancer and normal Gene Expression Profiling and Interactive Analyses. Nucleic Acids Res. 45, W98–W102. 10.1093/nar/gkx247 28407145 PMC5570223

[B40] Tasleem M. Alrehaily A. Almeleebia T. M. Alshahrani M. Y. Ahmad I. Asiri M. (2021). Investigation of Antidepressant Properties of Yohimbine by Employing Structure-Based Computational Assessments. Cimb 43 (3), 1805–1827. 10.3390/cimb43030127 34889886 PMC8929124

[B41] Tullemans B. M. E. Heemskerk J. W. M. Kuijpers M. J. E. (2018). Acquired Platelet Antagonism: Off-Target Antiplatelet Effects of Malignancy Treatment with Tyrosine Kinase Inhibitors. J. Thromb. Haemost. 16 (9), 1686–1699. 10.1111/jth.14225 29975003

[B42] Verhoeven Y. Tilborghs S. Jacobs J. De Waele J. Quatannens D. Deben C. (2020). The Potential and Controversy of Targeting STAT Family Members in Cancer. Semin. Cancer Biol. 60, 41–56. 10.1016/j.semcancer.2019.10.002 31605750

[B43] Wu H.-T. Liu J. Li G.-W. Shen J.-X. Huang Y.-T. (2017). The Transcriptional STAT3 Is a Potential Target, whereas Transcriptional STAT5A/5B/6 Are New Biomarkers for Prognosis in Human Breast Carcinoma. Oncotarget 8 (22), 36279–36288. 10.18632/oncotarget.16748 28422733 PMC5482654

[B44] Yang J. D. Hainaut P. Gores G. J. Amadou A. Plymoth A. Roberts L. R. (2019). A Global View of Hepatocellular Carcinoma: Trends, Risk, Prevention and Management. Nat. Rev. Gastroenterol. Hepatol. 16 (10), 589–604. 10.1038/s41575-019-0186-y 31439937 PMC6813818

[B45] Yang X. Tang Z. Zhang P. Zhang L. (2019). Research Advances of JAK/STAT Signaling Pathway in Lung Cancer. Zhongguo Fei Ai Za Zhi 22 (1), 45–51. 10.3779/j.issn.1009-3419.2019.01.09 30674393 PMC6348154

[B46] Zhang H. Liu H. Shen Z. Lin C. Wang X. Qin J. (2018). Tumor-infiltrating Neutrophils Is Prognostic and Predictive for Postoperative Adjuvant Chemotherapy Benefit in Patients with Gastric Cancer. Ann. Surg. 267 (2), 311–318. 10.1097/sla.0000000000002058 27763900

[B47] Zhao W. Li J. Chen M.-J. M. Luo Y. Ju Z. Nesser N. K. (2020). Large-Scale Characterization of Drug Responses of Clinically Relevant Proteins in Cancer Cell Lines. Cancer cell 38 (6), 829–843. 10.1016/j.ccell.2020.10.008 33157050 PMC7738392

[B48] Zhu X.-D. Sun H.-C. (2019). Emerging Agents and Regimens for Hepatocellular Carcinoma. J. Hematol. Oncol. 12 (1), 110. 10.1186/s13045-019-0794-6 31655607 PMC6815423

[B49] Zrieq R. Ahmad I. Snoussi M. Noumi E. Iriti M. Algahtani F. D. (2021). Tomatidine and Patchouli Alcohol as Inhibitors of SARS-CoV-2 Enzymes (3CLpro, PLpro and NSP15) by Molecular Docking and Molecular Dynamics Simulations. Int. J. Mol. Sci. 22 (19). 10.3390/ijms221910693 PMC8509278 34639036

