# Supplementary figures and images for: Lestaurtinib Has the Potential to Inhibit the Proliferation of Hepatocellular Carcinoma Uncovered by Bioinformatics Analysis and Pharmacological Experiments
Source: Front Cell Dev Biol. 2022 May 13;10:837428. doi: 10.3389/fcell.2022.837428 (PMC9136166; doi:10.3389/fcell.2022.837428)

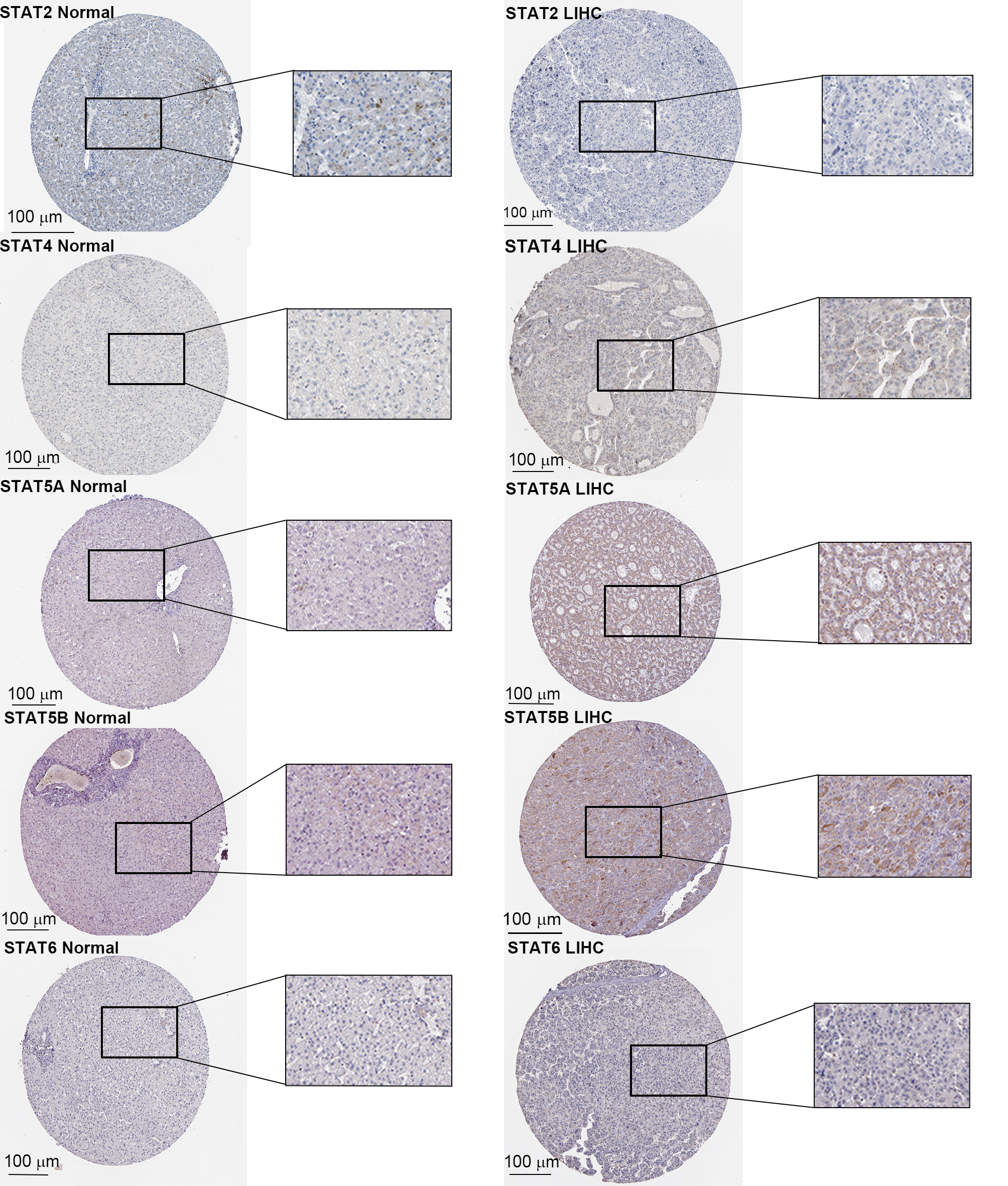

Supplement: Supplementary file 1 [file Image2.TIF]

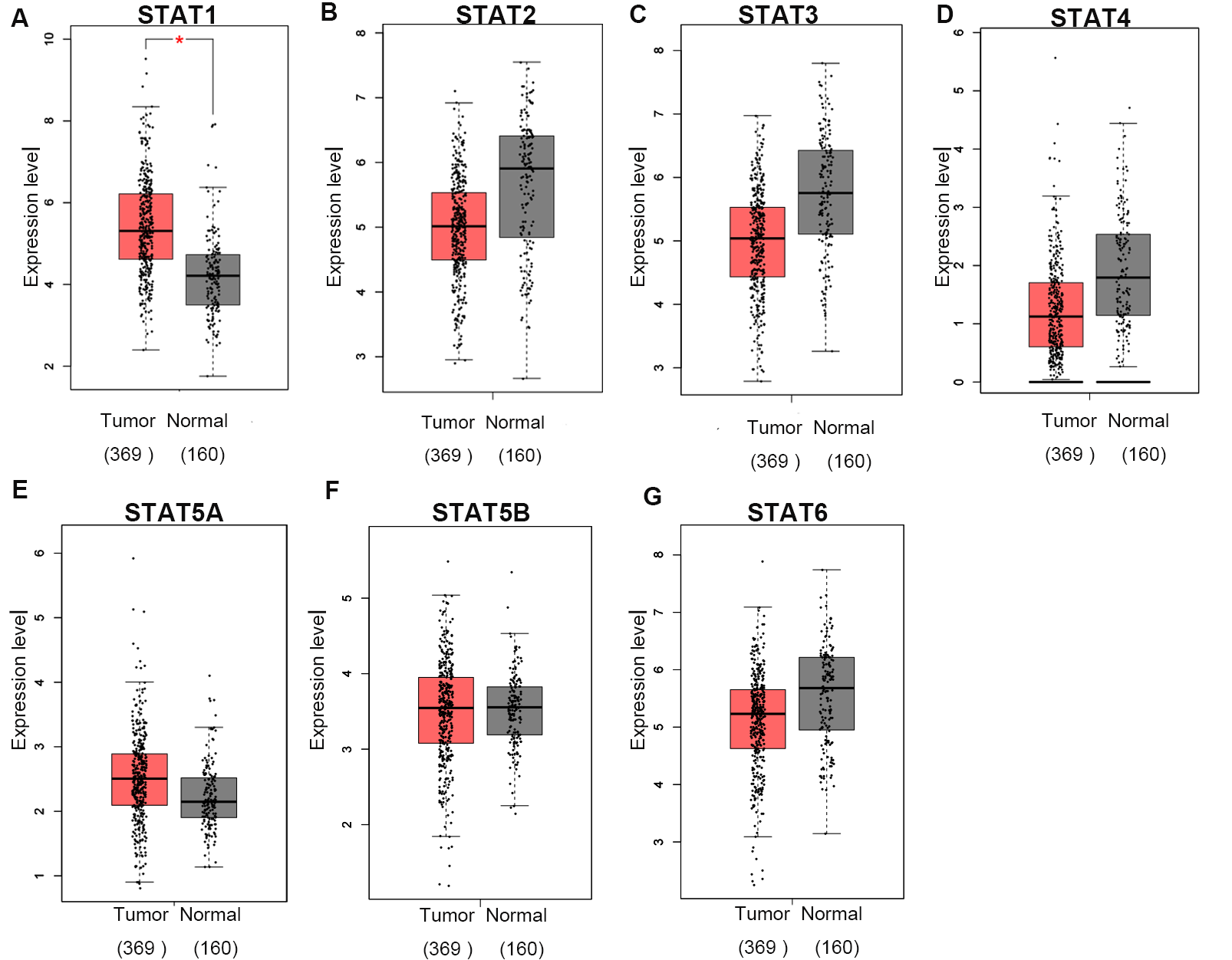

Supplement: Supplementary file 2 [file Image1.TIF]
